# Coated, Stabilized Enhanced-Efficiency Nitrogen Fertilizers: Preparation and Effects on Maize Growth and Nitrogen Utilization

**DOI:** 10.3389/fpls.2021.792262

**Published:** 2021-12-23

**Authors:** Zenglian Qi, Yuanjie Dong, Mingrong He, Maoying Wang, Yu Li, Xinglong Dai

**Affiliations:** ^1^College of Resources and Environment, Shandong Agricultural University, Taian, China; ^2^Agronomy College, Shandong Agricultural University, Taian, China

**Keywords:** enhanced-efficiency nitrogen fertilizer, natural rubber, urease inhibitor, nitrification inhibitor, maize yield, nitrogen use efficiency

## Abstract

Coated, slow/controlled release, or stabilized enhanced-efficiency nitrogen fertilizers (EENFs) are effective in improving nitrogen utilization efficiency (NUE) and crop yield. Better performance is expected from coated, stabilized EENFs where urease and nitrification inhibitors are treated in coated fertilizers. Firstly, five coated EENFs with different mass proportions of nature rubber (NR) in coating were prepared: CU0, CU1, CU2, CU3, CU4, and CU5 (0, 10, 20, 30, 40, and 50% of NR in coating). The controlled release performance of CU was tested by hydrostatic release test and the microstructure of controlled release urea, so as to screen the optimal addition ratio of NR (ER: NR = 7:3, CU3). Secondly, two coated, stabilized EENFs, CSU1 and CSU2, were prepared with natural rubber-modified epoxy resin (ER: NR = 7:3) as coating material. Seven treatments of different N fertilization were set up: CK (no N fertilization), urea, CU3, SU1, and SU2 (urease and nitrification inhibitors-treated urea fertilizers), CSU1 and CSU2 (urease and nitrification inhibitors-treated natural rubber-modified epoxy resin-coated urea fertilizers). Ammonia volatilization experiment and column leaching experiment showed that compared with conventional urea, NH_3_ volatilization loss was reduced by 20% and inorganic N leaching loss was reduced by 26% from CSU2, respectively. In the pot experiment, maize grain yield of 162.92 and 206.96 g/pot was achieved by CSU1 and CSU2, respectively, 41 and 79%, respectively, higher than that achieved by conventional urea. SUs treatments were more effective than conventional urea treatment in improving maize grain yield and NUE, but lower than in CSUs. The NUE, nitrogen fertilizer apparent utilization efficiency, partial factor productivity of applied N, and nitrogen utilization efficiency were 46, 30, 46, and 32%, respectively, higher in CSU1 and 58, 62, 58, and 29%, respectively, higher in CSU2 than in the conventional urea treatment. Compared with CSU1, CSU2 had better agronomic effectiveness with a higher NUE. It is recommended that urease and nitrification inhibitors be sandwiched between urea prill and the coating for preparation of novel, environmentally friendly coated, stabilized EENFs with high agronomic effectiveness, high NUE, and low N loss.

## Graphical Abstract

Synthesis process, inspection and application of EENFs.

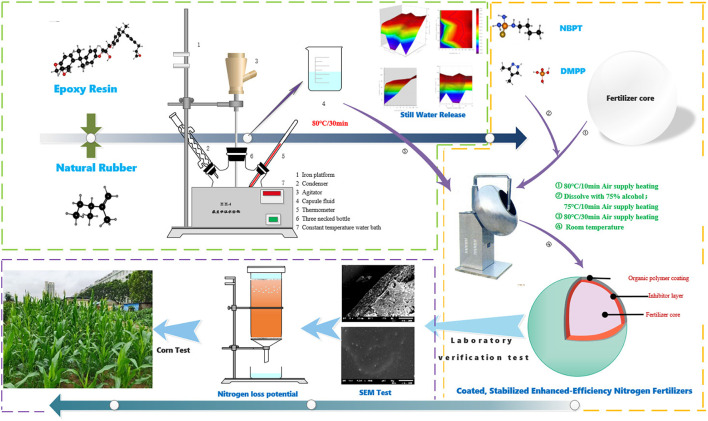

## Introduction

Nitrogen (N) is the most important mineral element for crop growth (Nasima, 2011; Santos et al., 2020) and greatly influences the yield and quality of agricultural products (Savin et al., 2019). However, soil N supply is limited and N fertilizers are commonly applied to maintain crop yield levels (Santos et al., 2020; Khampuang et al., 2021). The improvement of soil fertility requires the use of fertilizers, which now play an important role in agricultural productivity and food security. Globally, experience has shown that fertilization is the most effective way of increasing food production (Araujo et al., 2017). According to the Food and Agriculture Organization of the United Nations, since 1978, the total consumption of agricultural fertilizers has been 1.49 billion tons, of which N fertilizer accounts for 63% of the total consumption of agricultural fertilizers in China (FAO, 2019). Urea (46% N) is currently the main N fertilizer synthesized in China, accounting for more than 50% of the total N fertilizers produced (Li et al., 2015).

However, urea is usually directly spread on soil surface before irrigation in China (Ju and Gu, 2014), which leads to rapid hydrolysis of the fertilizer. Studies have shown that more than 50% of the applied N can be lost via surface runoff, leaching, and volatilization, resulting in low N use efficiency (NUE) (Azeem et al., 2014; Huang et al., 2017; Pereira et al., 2017). In 2015, NUE in China, India, United States, and the world was 30, 21, 41, and 35%, respectively (Omara et al., 2019). In China, more than 35 million tons of chemical N fertilizers were used in agricultural production in 2012, of which, at least 60% were lost to the environment (Huang et al., 2015), causing a series of environmental problems such as air pollution, water pollution, and soil degradation (Geng et al., 2016; Alhaj Hamoud et al., 2019b; Xiao et al., 2019).

According to the plant nutrient theory, optimal growth can be achieved if the nutrients were supplied based on the relative growth rate of crops. Developing coated controlled-release urea or urease/nitrification inhibitors is crucial, which can synchronize nutrient release rates for requirement patterns of the crop in the natural field.

Coating urea prills with organic polymers can prevent urea prills from direct contact with water and soil, thus effectively slowing down urea dissolution, reducing N loss, and improving NUE (Ji et al., 2013; Azeem et al., 2014; Geng et al., 2016). Such coated urea fertilizers are controlled release enhanced-efficiency N fertilizers (EENFs). Ji et al. (2017) reported that basal application of controlled-release EENFs significantly increased maize yield and NUE while reducing N loss as compared with conventional urea at the same N application rate.

Recent studies showed that combined application of urease inhibitor and urea reduces ammonia (NH_3_) volatilization loss (Ji et al., 2014; Li et al., 2015; Santos et al., 2020), increases crop yield, and improves NUE (Li et al., 2015). Urease inhibitors are the general term for a class of substances that have inhibitory effects on soil urease activity. Therefore, urease inhibitors can delay urea hydrolysis which is catalyzed by urease (Ji et al., 2014). N-(n-Butyl) thiophosphoric triamide (NBPT) is one of the most widely used urease inhibitors (Adotey et al., 2017). It is a structural analog of urea acting with mixed inhibition on urease activity (Zanin et al., 2015).

Combined application of nitrification inhibitor and urea effectively inhibits the activity of ammonia (NH_3_) oxidizing bacteria, delays the biological oxidation of NH_4_^+^ to NO_3_^–^ (nitrification process), reduces NO_3_^–^ loss and N_2_O emission, and improves NUE (Kawakami et al., 2012). 3,4-dimethylpyrazole phosphate (DMPP) is a commonly used nitrification inhibitor. It has the characteristics of high efficiency, non-toxicity, high stability, and high specificity. Souza et al. (2019) reported that the combined application of urea and DMPP would mitigate N_2_O emission. Abalos et al. (2014) recommended that nitrification and urease inhibitors be used to increase crop productivity and NUE.

Urea fertilizers treated with urease inhibitor and/or nitrification inhibitor are stabilized EENFs where urea is stabilized from rapid hydrolysis and/or nitrification. However, urease and nitrification inhibitors are subjected to adsorption, fixation, and degradation in soil (Engel et al., 2015; Santos et al., 2020), which greatly affect their action time and inhibitory efficiency. It is speculated that when coated, stabilized EENFs would present better agronomic effectiveness and higher NUE. Epoxy resin (ER) is a good coating material for controlled release fertilizers (Li et al., 2020b). However, it does not readily degrade and its accumulation in soil could be an environmental concern. Natural rubber (NR, mainly cis-1,4-polyisoprene) is a natural, green, and renewable material. In this study, ER was first modified with NR at different mass proportions for preparation of NR-modified ER-coated EENFs (referred to as CUs). Optimal NR mass proportion was chosen based on the N release characteristics of CUs. Then, two coated, stabilized EENFs (referred to as CSUs) were prepared with NR at optimal proportion in coating and urease and nitrification inhibitors treated using two methods. Nitrogen loss potential of the CSUs was evaluated with a NH_3_ volatilization experiment and a column leaching experiment, and agronomic effectiveness of the CSUs was evaluated with a pot experiment. Results from this study will provide a theoretical basis and technical support for the development of more environmentally friendly and more efficient EENFs.

## Materials and Methods

### Preparation of CUs

Firstly, NR-modified ER was prepared by mixing NR with ER at mass proportions of 0, 10, 20, 30, 40, and 50% in a three-necked flask equipped with a stirrer, a condenser, and a thermometer. The flask was immersed in water bath at 80°C for 30 min to obtain liquefied NR-modified ER.

Secondly, 1 kg urea (2–4 mm, 46% N) was loaded into a rotary drum blender (WKY-400, China) and preheated at 80 ± 2°C for 10 min. Then, 10.0 g liquefied NR-modified ER were poured onto the rotating urea prills and cured for 8 min. This step was repeated three times so that a total of 40.0 g liquefied NR-modified ER were used. Six CUs, i.e., CU0, CU1, CU2, CU3, CU4, and CU5, were prepared with NR mass proportion in coating of 0, 10, 20, 30, 40, and 50%, respectively.

### Microstructure Analyses of the CUs and Their Coatings

The surface and cross-sectional morphologies of the CUs were observed with a scanning electron microscope (SEM; JSM-6610LV, Japan) (Tian et al., 2019).

### Characterization of N Release From the CUs

A nitrogen release experiment was conducted to learn the appropriate mass proportion of NR for ER modification. Nitrogen release characteristics of the CUs were evaluated using the national standard method GB/T 23348-2009 (GAQS, IQPRC, SA, 2009). Briefly, 10.0 g CU were placed into a 250-ml glass bottle containing 200 ml distilled water at 25°C. Nitrogen concentration in the solution was determined using the Kjeldahl (1883) method after 1, 3, 5, 7, 10, 14, 28, 42, 56, and 84 days until cumulative N release rate was ≥80%, which is a common benchmark for complete release (Yang et al., 2013). Three replicates were set up for each CU. The CU with the best N release performance would be identified according to the controlled release period and its NR proportion in coating would be adopted in the subsequent preparation of CSUs.

### Preparation of Stabilized EENFs and CSUs

In order to test the effect of different formulations of inhibitor and urea on inhibitor activity, two types of uncoated, stabilized EENFs (referred to as SUs) were prepared: SU1 and SU2. The first type, SU1, was prepared by mixing 1 kg urea thoroughly with 1.15 g NBPT and 2.30 g DMPP. The preparation procedure of SU2 was similar to that of the CUs. Briefly, 1.15 g NBPT and 2.30 g DMPP were dissolved in 80 ml 75% ethanol solution. After 1 kg urea was loaded into a rotary drum blender (WKY-400, China) and preheated at 80 ± 2°C for 10 min, 20 ml of the NBPT and DMPP containing ethanol solution was poured onto the rotating urea prills and cured for 8 min. This step was repeated three times and SU2 was obtained.

Two CSUs (i.e., CSU1 and CSU2) were prepared in the same way as the CUs except that for CSU1, the first 10 g liquefied NR-modified ER contained 1.15 g NBPT and 2.30 g DMPP, and for CSU2, SU2 was used instead of urea. That is, NBPT and DMPP were homogeneously distributed in the innermost layer of the coating in CSU1, whereas they were sandwiched between the coating and the urea in CSU2. The surface and cross-sectional morphologies of the CSUs were observed with a SEM (JSM-6610LV, Japan) (Tian et al., 2019).

### Evaluation of the N Loss Potential of the SUs and CSUs

Two experiments were conducted to evaluate the N loss potential, including volatilization and leaching loss, of the SUs and CSUs.

The soil used in this study, including these two experiments and the pot experiment described later, was collected from the experimental station of the College of Resources and Environment, Shandong Agricultural University, China. The basic physical and chemical properties of the soil are shown in Table 1.

**TABLE 1:** Basic physical and chemical properties of the soil used in this study.

Soil classification	Soil texture	pH	EC (μS/cm)	SOM (g/kg)	Available N (mg/kg)	Total N (g/kg)	Available P (mg/kg)	Available K (mg/kg)
Brown earth	Clay loam	6.5	145.04	10.1	38.5	0.862	22.75	107.11

For NH_3_ volatilization loss evaluation, urea, CU3, the SUs, and the CSUs were each mixed thoroughly with 250 g soil at 0.6 g N/kg dry soil in plastic boxes. In addition, boxes containing only 250 g soil without fertilizer (CK) were also prepared. After soil moisture was adjusted to 60% of field water holding capacity with distilled water, a Petri dish containing 10 ml 3% boric acid indicator solution was placed on the soil as a trap for volatilized NH_3_. The boxes were sealed and incubated at 25°C in the dark. The boric acid traps were replaced with new ones at regular intervals during the 40 days of incubation and titrated with sulfuric acid standard solution (0.005 mol/l) for NH_3_ quantification (Zhou, 2017).

A column leaching experiment was conducted for evaluation of ammonium and nitrate leaching loss potential of the CU3, SUs, and CSUs. PVC columns (6.0 cm in diameter and 15.0 cm in height) were first packed with a thin layer of quartz sand, then 2000 g soil, and finally a thin layer of quartz sand again. The SUs and CSUs were each mixed thoroughly with the top 5 cm of soil at 0.6 g N/kg dry soil. Columns without fertilizer added were also set up as control (CK). Distilled water was added to saturate the soil for 24 h. Then, 100 ml distilled water was added to the column every 4 days for a total of 1000 ml water in 40 days. Leachate was collected for NH_4_^+^-N and NO_3_^–^-N determination using an AA3 continuous flow analyzer (BL-TECH, Germany). Soil inorganic N content was calculated as the sum of NH_4_^+^-N and NO_3_^–^-N.

### Pot Experiment

The pot experiment adopted a completely randomized block design. Seven treatments of different N fertilizations were set up: CK (no application of N fertilizer), urea, CU3, SU1, SU2, CSU1, and CSU2. Plastic pots (lower diameter 23.0 cm, upper diameter 35.0 cm, and height 43.5 cm) were used, and 15 kg of soil were put in each pot. The N fertilizers were applied at 0.15 g N/kg dry soil (equivalent to 337.5 kg N/ha). Calcium superphosphate was used as phosphorus fertilizer at 0.1 g P_2_O_5_/kg dry soil (equivalent to 225 kg P_2_O_5_/ha) and potassium chloride as potassium fertilizer at 0.1 g K_2_O/kg dry soil (equivalent to 225 kg P_2_O_5_/ha). The phosphorus and potassium fertilizers, SUs, and CSUs were applied as basal fertilizers in the subsurface soil layer, while urea was split-applied as basal fertilizer (50%) and topdressing (50%, at the jointing stage). Five seeds of maize (Zea mays Ziyu 2) were sown in each pot on June 10, 2020 and thinned to one seedling at the 5-leaf stage.

At the seedling (June 20), jointing (July 10), tasseling (August 10), flowering (August 17), and mature (September 22) stages of maize, three pots were randomly taken from each treatment. Plant heights were measured. After maize roots were removed, fresh soil samples were taken and extracted with 1 mol/l KCl for determination of NH_4_^+^-N and NO_3_^–^-N with an AA3 continuous flow analyzer (BL-TECH, Germany). Urease activity was measured by the sodium phenate-sodium hypochlorite colorimetric method (Santos et al., 2020) using air-dried soil (<1 mm). Maize was harvested on October 22. Ears per plant, grains per ear, and 100-grain weight were recorded. Plant and grain samples were first oven-dried at 105 °C for 30 min and then at 75°C to constant weight. The samples were digested with H_2_SO_4_/H_2_O_2_ and N content was determined by the Kjeldahl (1883) method. Maize grain yield, N utilization efficiency (NUTE), partial factor productivity of applied N (NPFP), N fertilizer apparent utilization efficiency (NFUE), and N use efficiency (NUE) were calculated as follows:


$$\begin{array}{l} \begin{array}{lll} \text{Grain yield } & = & \text{ plants per pot }\times \text{ ears per plant }\times \text{ grains per ear }\\ & & \times \text{ 100-grain weight/100}. \end{array}\\ \begin{array}{l} \text{NUTE = grain yield/N accumulation in aboveground parts}. \end{array}\\ \begin{array}{l} \begin{array}{l} \text{NPFP = grain yield/total N supplied by fertilizer}. \end{array} \end{array}\\ \begin{array}{lll} \text{NFUE } & = & \;(\text{N accumulation in aboveground parts of fertilization }\\ & & \text{treatment --N accumulation in aboveground parts}\\ & & \text{ of CK})\text{/total N supplied by fertilizer}. \end{array}\\ \begin{array}{l} \text{NUE = grain yield/total N supplied by soil and fertilizer}. \end{array} \end{array}$$

Soil apparent nitrification rate was calculated as:


$$\begin{array}{lll} \text{Soil apparent nitrification rate }(\%)\; & = & \;NO_{3}{}^{-}-N/(NH_{4}{}^{+}-N\\ & & +\;NO_{3}{}^{-}-N)\;\times \;100. \end{array}$$

### Statistical Analysis

Microsoft Excel 2016 was used for data processing and Origin 2021 software was used for figure drawing. Data were subjected to analysis of variance (ANOVA) using IBM SPSS Statistics 21.0 and means were separated by Duncan’s multiple range test (*P* < 0.05).

## Results

### Microstructure of the CUs

**FIGURE 1:**
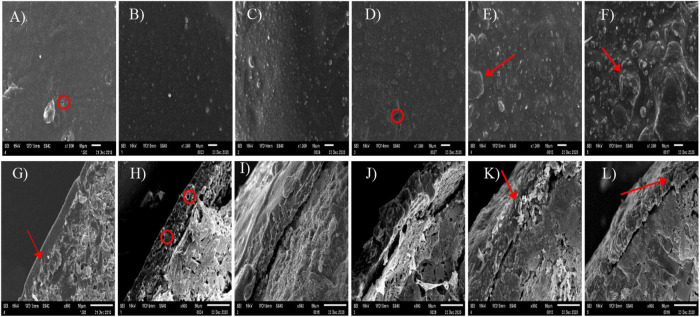
Surface **(A–F)** and cross sectional **(G–L)** scanning electron microscope (SEM) images of the prepared natural rubber-modified epoxy resin-coated urea fertilizers (CUs). Natural rubber mass proportions in coating were 0% (CU0, the 1st column), 10% (CU1, the 2nd column), 20% (CU2, the 3rd column), 30% (CU3, the 4th column), 40% (CU4, the 5th column), and 50% (CU5, the 6th column).

The surface and cross-sectional morphologies of the CUs are shown in Figure 1. The surface of the CUs became rougher with more NR in the coating. Pin holes of various sizes were observed on the surface of CU0 (Figure 1A). There were tiny bumps on the surface of CU3 (Figure 1D), whereas the surfaces of CRU4 and CRU5, the latter in particular, showed sheet structure (Figures 1E,F). The coating of CU0 displayed a compact structure, whereas that of CU1 was porous (Figures 1G,H). Loose sheet structure of the coating became more prominent with more NR (Figures 1I–L).

### Nitrogen Release Characteristics of the CUs

**FIGURE 2:**
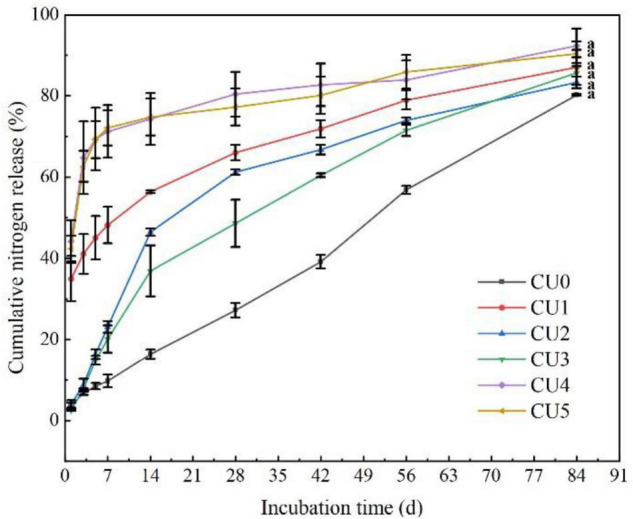
Nitrogen release curves of the natural rubber-modified epoxy resin-coated urea fertilizers (CUs). Natural rubber mass proportions in coating were 0% (CU0), 10% (CU1), 20% (CU2), 30% (CU3), 40% (CU4), and 50% (CU5).

Nitrogen was released rapidly from CU4 and CU5, with first-day release of 44 and 42%, respectively (Figure 2). After only 7 days of incubation, over 70% of total N had been released from the two CUs, and after 28 and 42 days, 80% of total N had been released from CU4 and CU5, respectively. In contrast, N release from CU2 and CU3 was much slower in the early stage of incubation, with merely approximately 15% being released in the first week. The N release period of CU2 and CU3 was 75 and 73 days, respectively, comparable to that of CU0 (84 days). For CU1, over 30% of total N was released in the first day of incubation, nearly 50% was released in the first 7 days, and 80% was released in 61 days. In a word, of the five NR-modified ER-coated EENFs, CU2 and CU3 presented the most comparable N release performance to that of CU0. Their first-day N release rates were far lower than 15% and their N release periods were approximately two and a half months, well meeting the requirement for a controlled release N fertilizer stated in the national standard method GB/T 23348-2009. The N release characteristics of the CUs were closely related to the structure of their coatings. The rapid N release from CU4 and CU5 is attributed to the loose sheet structure of their coatings, whereas the rapid N release from CU1 is due to the porous structure of its coating (Figure 1). Both loose sheet and porous structures are favorable for the diffusion of water through the coating and subsequent diffusion of N solution out to the soil (i.e., N release). Considering that the N release pattern of CU3 was more similar to that of CU0 and that a smaller proportion of ER would be better for the environment, the NR mass proportion of 30% in coating was adopted for the subsequent preparation of CSUs.

### Nitrogen Loss Potential of the SUs and CSUs

**FIGURE 3:**
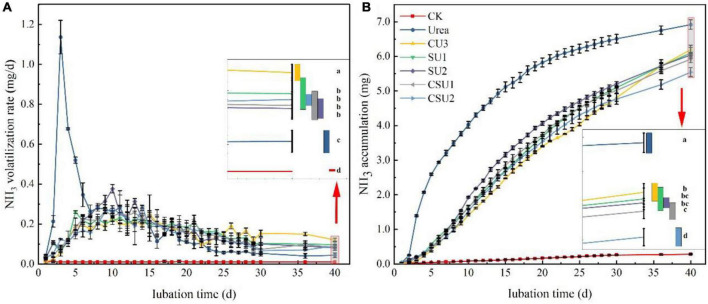
Ammonia volatilization rate **(A)** and accumulative volatilization **(B)** of the treatments with application of conventional urea, the natural rubber-modified epoxy resin-coated urea fertilizers (CU3), urease and nitrification inhibitors treated urea (SU1 and SU2), and inhibitors treated natural rubber-modified epoxy resin-coated urea (CSU1 and CSU2). CK: no urea was applied.

A large amount of NH_3_ volatilized from urea was measured in the first week of incubation, with peak volatilization of 1.13 mg NH_3_-N at day 3 (Figure 3A), indicating rapid hydrolysis of conventional urea after applied to the soil. Ammonia volatilization in the conventional urea treatment decreased continuously from day 4 to 9, fluctuated in day 10–19, and then decreased continuously again, with daily volatilization lower than those of the CU3, SU, and CSU treatments from day 16 on. In contrast, NH_3_ volatilization from the CU3, SU, and CSU treatments, which mainly occurred during days 7–17, was small during the entire incubation period, with even the peak daily volatilization smaller than 0.4 mg NH_3_-N. The NH_3_ volatilization in the CU3 reached the peak on the 14th day, which was 4 days later than the peak in the SU2. The NH_3_ volatilization rate of EENFs was higher than that of conventional urea during days 23–40. On the 40th day, the NH_3_ volatilization rate of CU3 was significantly higher than that of SUs and CSUs. During the cultivation stage, the NH_3_ volatilization accumulation of conventional urea was higher than that of EENFs (Figure 3B). Compared with the conventional urea treatment, the NH_3_ volatilization was significantly decreased by 10, 12, 13, 14, and 20% in CU3, SU1, SU2 CSU1, and CSU2, respectively. In addition, NH_3_ volatilization from the CSU2 treatment was significantly lower than those from the CU3, CSU1, and SUs treatments, whereas there were no significant differences between CSU1 and SUs.

**FIGURE 4:**
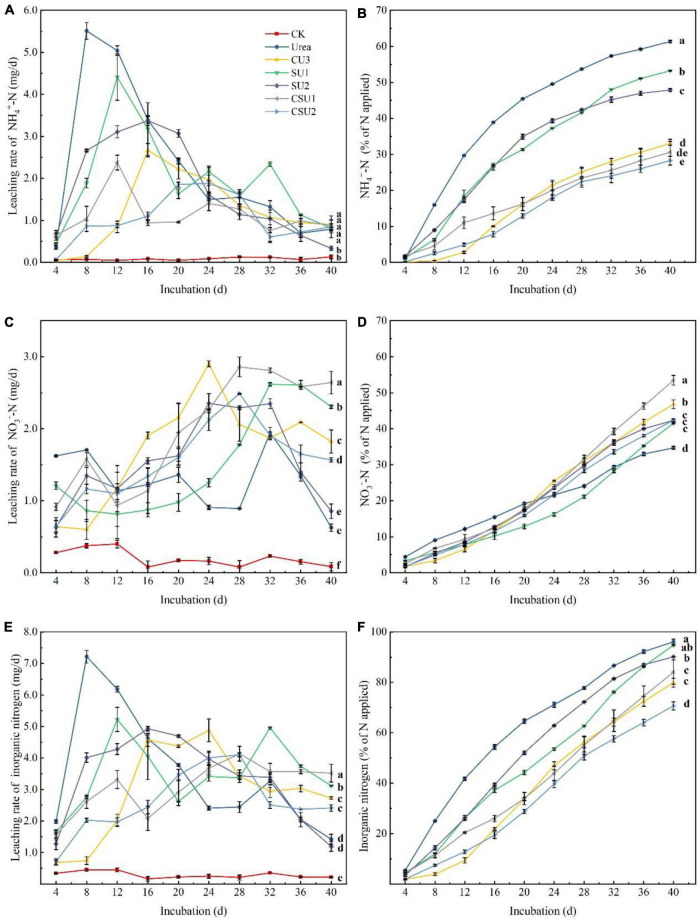
Leaching rates and cumulative leached amounts of NH_4_^+^-N and NO_3_^–^-N from urea, the natural rubber-modified epoxy resin-coated urea fertilizers (CU3), urease and nitrification inhibitors treated urea (SU1 and SU2), and the coated and urease and nitrification inhibitors treated urea fertilizers (CSU1 and CSU2). **(A)** NH_4_^+^-N leaching rate; **(B)** Cumulative leached NH_4_^+^-N; **(C)** NO_3_^–^-N leaching rate; **(D)** Cumulative leached NO_3_^–^-N; **(E)** Inorganic N leaching rate; **(F)** Cumulative leached inorganic N.

Leaching loss of NH_4_^+^-N, NO_3_^–^-N, and inorganic N from the CU3, SUs, CSUs, and conventional urea are shown in Figure 4. The peak NH_4_^+^-N leaching rate occurred at day 8, 16, 12, 16, 12, and 24 in the urea, CU3, SU1, SU2, CSU1, and CSU2 treatments, respectively (Figure 4A). Compared with the urea treatment, the peak NH_4_^+^-N leaching rate decreased by 52, 20, 39, 57, and 66% in SU1, SU2, CSU1, and CSU2, respectively. Throughout the leaching experiment, NH_4_^+^-N was the major inorganic N in the leachates from the conventional urea, SU1, and SU2 treatments. In addition, the leached amount of NH_4_^+^-N from the CSUs treatments was significantly lower than those from the SU1 and SU2 treatments, whereas there were no significant differences between CSU1 and CSU2 (Figure 4B). Furthermore, the leached amount of NH_4_^+^-N from the SU treatments was significantly less than that from the conventional urea treatment, and the leached amount of NH_4_^+^-N from the CSU treatments was significantly less than that from the SU treatments. In the 40 days of incubation, the cumulative leached amount of NH_4_^+^-N was 46, 13, 22, 50, and 54% lower from CU3, SU1, SU2, CSU1, and CSU2, respectively, than from conventional urea.

The leaching dynamics of NO_3_^–^-N was different from that of NH_4_^+^-N. It can be seen from Figure 4C that the NO_3_^–^-N leaching rate of conventional urea was higher than those of the CU3, SUs, and CSUs in the first 12 days. This is due to the rapid hydrolysis of urea, which generated a large amount of NH_4_^+^-N, the substrate of nitrification, and accelerated the production of NO_3_^–^-N. The NO_3_^–^-N leaching rate in the conventional urea treatment decreased from day 20 to 28 and was lower than those in the SU and CSU treatments during day 24–28. In the 40 days of incubation, the cumulative leached amount of NO_3_^–^-N was 38, 47, 42, 43, 53, and 42% from U, CU3, SU1, SU2, CSU1, and CSU2, respectively (Figure 4D). The decrease was attributed to the rapid hydrolysis of urea and leaching of NH_4_^+^-N out from the column, which led to weak nitrification and low NO_3_^–^-N in the later stage. In contrast, in the SU and CSU treatments, NO_3_^–^-N leaching rate was low in the early stage and increased in the later stage. This is attributed to the presence of DMPP, which inhibits nitrification. With the gradual dissolution of DMPP, its inhibition on nitrification became weaker and more NH_4_^+^-N was converted to NO_3_^–^-N.

The leaching loss of soil inorganic N was calculated (Figure 4F). After the incubation, the leaching loss of soil inorganic N was significantly reduced in CU3, SU2, CSU1 and CSU2 by 17, 6, 13, and 26%, respectively, compared with the conventional urea treatment. There was no significant difference in the leached amount of inorganic N between the SU1 and conventional urea treatments. In terms of leached amount of applied N, the fertilizers were in the order of: conventional urea and SU1 > SU2 > CSU2 and CU3 > CSU2.

### Agronomic Effectiveness of the SUs and CSUs

#### Changes in Soil Inorganic N and Apparent Nitrification Rate During Maize Growth

**FIGURE 5:**
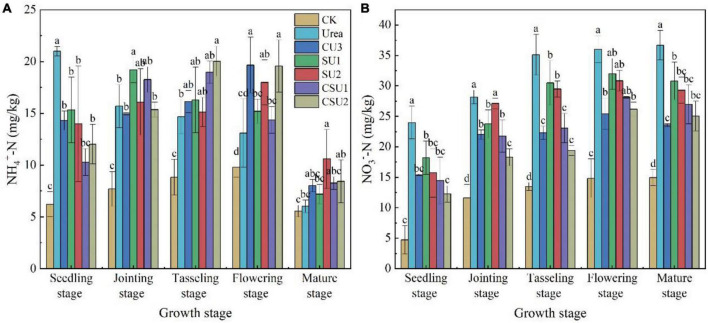
Soil NH_4_^+^-N **(A)** and NO_3_^–^-N **(B)** contents at different growth stages of maize in the different treatments of the pot experiment. CK: no N fertilizer was applied; Urea: conventional urea; CU3: the natural rubber-modified epoxy resin-coated urea fertilizers; SU1 and SU2: urease and nitrification inhibitors treated urea fertilizers; CSU1 and CSU2: urease and nitrification inhibitors treated natural rubber-modified epoxy resin-coated urea fertilizers. Different letters indicate significant differences between treatments for a same growth stage (*P* < 0.05).

In the conventional urea treatment, soil NH_4_^+^-N content decreased with time during maize growth (Figure 5A). It was over 20 mg/kg at the seedling stage and decreased to slightly more than 15 mg/kg at the jointing stage and lower than 15 mg/kg at the tasseling and flowering stages. In contrast, in the CU3, SU and CSU treatments, soil NH_4_^+^-N content was low at the seedling stage, 15 mg/kg or even lower, increased at the jointing, tasseling, and flowering stages, and then decreased significantly at the mature stage. In the conventional urea treatment, the high NH_4_^+^-N content at the early growth stages of maize may exceed plant demand and lead to N loss to the environment, resulting in low NUE and eutrophication of waters, whereas the low NH_4_^+^-N content in the maize rapid growth stages may constrain plant growth, leading to low crop yield. In contrast, the temporal changes of NH_4_^+^-N content in the SU and CSU treatments presented a plant demand-synchronized pattern, which not only meets plant nutrient demand but also minimizes N loss.

The NO_3_^–^-N content increased with time in the conventional urea treatment, whereas it increased with time until the flowering stage and decreased a little bit at the mature stage in the other treatments (Figure 5B). In addition, it was always higher in the conventional urea treatment than in the other fertilization treatments, demonstrating the effectiveness of nitrification inhibitor in inhibiting the oxidation conversion of NH_4_^+^-N to NO_3_^–^-N. The results of this experiment validated the effects of three different fertilizers: coated (CU3), inhibitor (SU_*S*_) and combined coating and inhibitor (CSU_*S*_) on soil N transformation. The combined effect of coating and inhibitor was stronger than coating alone or adding inhibitor to increase the nutrient content in the critical period of maize growth and reduce the NO_3_^–^-N in the whole growth stage.

The apparent nitrification rate in the conventional urea treatment was high during maize growth (Table 2), resulting in high soil NO_3_^–^-N content (Figure 5B). At the jointing stage of maize, the apparent nitrification rate in CU3, SU1, SU2, CSU1, and CSU2 was 7, 14, 2, 16, and 16%, respectively, lower than that in the conventional urea treatment. At the tasseling stage, the apparent nitrification rate in the CSU treatments was on average 26 and 21% lower than that in the SU treatments and conventional urea treatment, respectively. At the flowering and mature stage of maize, the apparent nitrification rate in CSU2 was 21 and 13% lower than that in conventional urea. From the tasseling stage to the maturity stage, the apparent nitrification rate of CU3 was lower than that of SUs treatment. The low apparent nitrification rate in the CSU treatments was due to the presence of the NR-modified ER coating and the biochemical inhibitors (NBPT + DMPP). The coating and inhibitors not only physically slowed down the dissolution of urea, but also chemically and biologically slowed down the hydrolysis and oxidation conversion of urea, reducing NH_3_ volatilization loss at the early stage of fertilization and NO_3_^–^-N leaching loss at the later stage. The coating and inhibitors (CSU) not only physically slowed down the dissolution of urea, but also chemically and biologically slowed down the hydrolysis and oxidation conversion of urea, reducing NH_3_ volatilization loss at the early stage of fertilization and NO_3_^–^-N leaching loss at the later stage.

**TABLE 2:** Soil apparent nitrification rate (%) at different growth stages of maize in the different treatments.

Treatment	Seedling stage	Jointing stage	Tasseling stage	Flowering stage	Mature stage
CK	41.6 ± 15.6b	60.2 ± 7.5ab	60.5 ± 2.8bc	59.8 ± 7.2bc	72.8 ± 3.7c
Urea	53.2 ± 3.4ab	64.3 ± 3.1a	70.6 ± 0.5a	73.5 ± 4.5a	85.7 ± 2.7a
CU3	51.9 ± 1.6ab	59.6 ± 1.0ab	58.0 ± 2.1cd	56.5 ± 3.7c	74.6 ± 1.5c
SU1	54.5 ± 3.0ab	55.3 ± 3.7b	65.2 ± 5.1ab	67.7 ± 3.0ab	81.1 ± 3.1ab
SU2	52.4 ± 8.2ab	63.1 ± 4.5a	66.1 ± 3.2ab	62.3 ± 6.9bc	75.8 ± 2.5c
CSU1	57.8 ± 7.0a	54.3 ± 3.0b	54.8 ± 3.4d	66.2 ± 2.1ab	76.4 ± 2.7bc
CSU2	50.6 ± 6.4ab	54.3 ± 2.8b	49.3 ± 2.5e	57.4 ± 2.1c	74.6 ± 2.5c

*CK: no N fertilization; urea: conventional urea was applied; CU3: the natural rubber-modified epoxy resin-coated urea fertilizers; SU1 and SU2: urease and nitrification inhibitors treated urea fertilizers. CSU1 and CSU2: urease and nitrification inhibitors treated natural rubber-modified epoxy resin-coated urea fertilizers. Different letters indicate significant differences between treatments for a same growth stage (P < 0.05).*

#### Urease Activity

**FIGURE 6:**
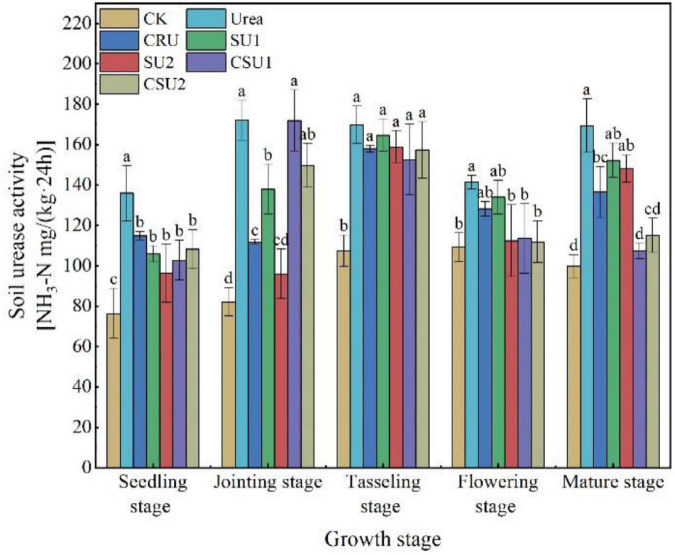
Soil urease activity at different growth stages of maize in the different treatments. CK: no N fertilization; urea: conventional urea; CU3: the natural rubber-modified epoxy resin-coated urea fertilizers; SU1 and SU2: urease and nitrification inhibitors treated urea fertilizers; CSU1 and CSU2: urease and nitrification inhibitors treated natural rubber-modified epoxy resin-coated urea fertilizers. Different letters indicate significant differences between treatments for a same growth stage (*P* < 0.05).

The urease activity was always higher in the conventional urea treatment than in the other treatments (Figure 6). Compared with the conventional urea treatment, soil urease activity in the CSU2 treatment was lower by 20, 13, 7, 20, and 32% at the seedling, jointing, tasseling, flowering, and mature stages, respectively. Soil urease activity in the SU treatments was lower than that in the CSU treatments at the seedling and jointing stages but was higher at the tasseling, flowering, and mature stages.

#### Plant Height and Grain Yield

**FIGURE 7:**
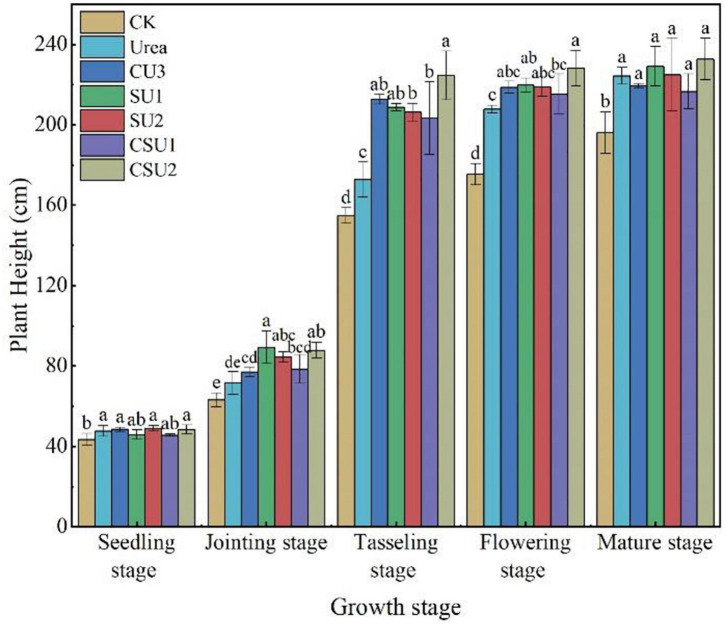
Maize plant height at different growth stages in different treatments. CK: no N fertilization; urea: conventional urea; CU3: the natural rubber-modified epoxy resin-coated urea fertilizers; SU1 and SU2: urease and nitrification inhibitors treated urea fertilizers; CSU1 and CSU2: urease and nitrification inhibitors treated natural rubber-modified epoxy resin-coated urea fertilizers. Different letters indicate significant differences between treatments for a same growth stage (*P* < 0.05).

Maize plant height in the CU3, SU, and CSU treatments increased more rapidly and was higher than that in CK and the conventional urea treatment at the jointing and tasseling stages (Figure 7). However, plant height in the CU3, SU, and CSU treatments was much smaller than that in CK and the conventional urea treatment at the flowering and mature stages, indicating that the N supply pattern in the CU3, SU, and CSU treatments could meet the N demand of maize in the transition from vegetative growth to reproductive growth of the maize.

The highest maize grain yield was obtained in CSU2, followed by CSU1, CU3, SU1, SU2, and urea (Table 3). Maize grain yield in the CU3, SU, and CSU treatments was 12–79% higher than that in the conventional urea treatment. The grain yield difference between the CSU treatments and the conventional urea treatment was significant. There was no significant difference in maize yield between CU3 and SU treatments. Though the grain yields in the SU treatments were higher than that in the urea treatment, the differences were not significant, indicating that direct exposure of the urea prills and inhibitors to the soil had greatly compromised the agronomic effectiveness of the urea fertilizer and the effectiveness of the inhibitors as well.

**TABLE 3:** Maize grain yield and yield formation factors.

Treatment	Grain number pot^–1^	100-grain weight (g)	Grain yield (g pot^–1^)
CK	345 ± 13d	30.84 ± 0.76e	106.38 ± 6.78e
Urea	373 ± 21cd	31.03 ± 0.59e	115.84 ± 6.36de
CU3	425 ± 31c	32.94 ± 0.72bc	139.90 ± 10.75c
SU1	401 ± 41c	33.97 ± 0.99ab	136.08 ± 9.74c
SU2	415 ± 15c	31.29 ± 0.36de	129.71 ± 3.52cd
CSU1	504 ± 48b	32.36 ± 0.66cd	162.92 ± 13.19b
CSU2	601 ± 28a	34.44 ± 0.45a	206.96 ± 11.85a

*CK: no N fertilization; urea: conventional urea was applied; CU3: the natural rubber-modified epoxy resin-coated urea fertilizers; SU1 and SU2: urease and nitrification inhibitors treated urea fertilizers; CSU1 and CSU2: urease and nitrification inhibitors treated natural rubber-modified epoxy resin-coated urea fertilizers. Different letters in a same column indicate significant differences at P < 5%.*

#### NUE, NFUE, NPFP, and NUTE

The different treatments had significantly different NUE, NFUE, NPFP and NUTE (Table 4). The highest NUE, NFUE, NPFP, and NUTE values were all obtained in CSU2, whereas the lowest values were obtained in the conventional urea treatment. The NUE, NFUE, NPFP, and NUTE values were 46, 30, 46, and 32%, respectively, higher (*P* < 0.05) in CSU1 and 58, 62, 58, and 29%, respectively, higher (*P* < 0.05) in CSU2 than in the conventional urea treatment. The highest NUE was obtained in CSU2, followed by CSU1, SU1, CU3, SU2, and urea, which was consistent with the change of maize yield. The NUE values confirm that stabilizing urea with urease and nitrification inhibitors is effective in improving its NUE and that coating can further improve its NUE, implying less N loss to the environment.

**TABLE 4:** Nitrogen use efficiency (NUE), nitrogen fertilizer apparent utilization efficiency (NFUE), partial factor productivity of applied N (NPFP), and nitrogen utilization efficiency (NUTE) of the different treatments.

Treatment	NUE (g g^–1^)	NFUE (%)	NPFP (g g^–1^)	NUTE (g g^–1^)
CK	-	-	-	-
Urea	41.1 ± 0.5f	38.6 ± 8.4b	54.4 ± 0.7f	56.3 ± 5.8c
CU3	47 ± 0.3d	44.1 ± 1.0b	62.2 ± 0.3d	60.5 ± 0.8c
SU1	49.8 ± 0.3c	46.2 ± 1.6b	65.9 ± 0.4c	62.9 ± 0.6c
SU2	44.9 ± 1.0e	45.3 ± 1.5b	59.4 ± 1.3e	64.3 ± 8.2bc
CSU1	59.7 ± 1.2b	48.6 ± 10.8b	79.1 ± 1.5b	74.2 ± 6.3a
CSU2	64.9 ± 0.9a	59.4 ± 1.2a	86.0 ± 1.2a	72.9 ± 0.3ab

*CK: no N fertilization; urea: conventional urea; CU3: the natural rubber-modified epoxy resin-coated urea fertilizers; SU1 and SU2: urease and nitrification inhibitors treated urea fertilizers; CSU1 and CSU2: urease and nitrification inhibitors treated natural rubber-modified epoxy resin-coated urea fertilizers. Different letters in a same column indicate significant differences between treatments at P < 5%.*

## Discussion

### Evaluation of the Controlled Release Mechanism of CUs and CSUs

Nitrogen requirement for maize during the growth stage followed an “S-shaped” curve (Zheng et al., 2017). Therefore, N supplement according to the different nutrient demands in each growth stage is particularly important for a high and stable yield of maize. Conventional quick-acting N fertilizer needs to be topdressing during the crop growth period. Advantageously, the basic application of controlled release EENFs can meet N demand during the crop growth stage and significantly improve the matching degree between soil N supply and crop nutrient demand (Geng et al., 2016; Xiao et al., 2019), promote crop yield and NUE. It was found that the N release performance of controlled release EENFs is related to the characteristics of coating material and thickness, as well as environmental factors such as soil temperature and moisture (Zhang et al., 2018). The difference in coating material characteristics is the direct factor affecting the N release of controlled release EENFs. An et al. (2021) reported that the nutrient release of coated fertilizers mainly has three stages, including the transport of water into the capsule, dissolution of fertilizers and release of the nutrients through coating materials. This experiment used natural rubber (NR)-modified ER as coating material to prepare coated EENFs. The results of the SEM (Figure 1D) showed that the surface of CU3 was smooth, without apparent protrusions, and the surface film holes were moderate. There were a few nutrient channels in the cross section of CU3. It is speculated that the controlled release mechanism of CU3 was consistent with the nutrient diffusion mechanism of conventional resin-coated urea. After coated controlled release EENFs were applied to the soil, as soil moisture penetrates into the membrane shell and dissolves the urea core, the osmotic pressure difference between the inside and outside of the membrane gradually increases. The pressure on the membrane shell changes the microstructure (the density of nutrient channels on the membrane shell increases), and the N was slowly released through the nutrient channel under the action of osmotic pressure. The hydrostatic release curve showed that the nutrient release process of CU3 could be roughly divided into two stages. (1) The first stage (1–7 days) is the slow-release period, in which the cumulative release of N was 20%; (2) The second stage (8–56 days) is the rapid release period, and the cumulative amount of N was 71%. The “N backward shift” phenomenon in hydrostatic release was consistent with high-yield maize still needed to absorb more N to meet crop material synthesis demand in the mid-late growth stage (Wang et al., 2010). The results demonstrated that the content of NR would reduce the controlled release performance as compared with CU0. However, controlling the amount of NR could reduce the amount of ER and reduce the pressure of resin on the soil environment without significantly changing the controlled-release performance of CU0.

**FIGURE 8:**
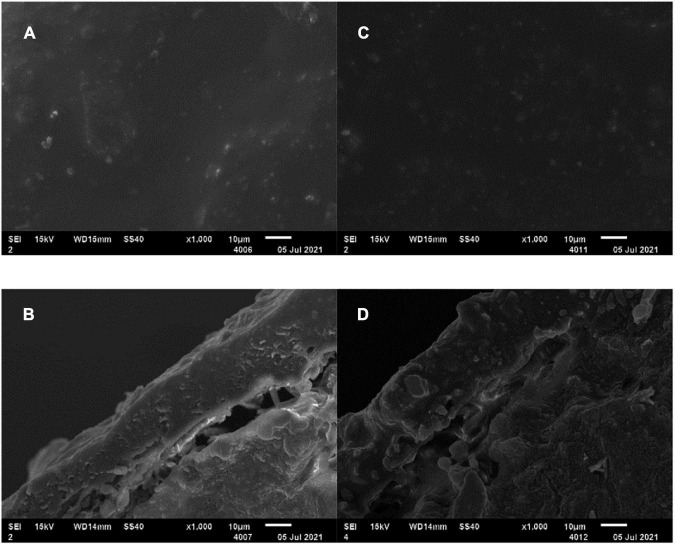
SEM of CSU1 and CSU2. **(A)** SEM of CSU1 surface (×1000); **(B)** CSU1 section electron microscope (×1000); **(C)** SEM of CSU2 surface (×1000); **(D)** CSU2 section electron microscope (×1000).

To strengthen the matching degree between crop nutrient requirements and soil nitrogen supply, NR mass proportion of 30% in coating was adopted in the subsequent preparation of two EENFs: CSU1 (inhibitors homogeneously distributed in the innermost layer of the coating) and CSU2 (inhibitors sandwiched between urea and coating). Theoretically, the slow dissolution of N and NBPT + DMPP could be controlled simultaneously to realize the dual regulation of conventional urea dissolution and transformation. Li et al. (2020a) reported that the slow-release periods of coated controlled-release urea for N, hydroquinone (HQ), and dicyandiamide (DCD) were 56, 42, and 14 days, respectively. However, because determination of NBPT and DMPP content requires high-performance liquid chromatography (HPLC) (Santos et al., 2020). Therefore, it is difficult to directly determine the activity of NBPT and DMPP in EENFs and the controlled release performance of the organic membrane shell on the inhibitor. Furthermore, there is uncertainty about the true DMPP and NBPT application rate with the coated fertilizers due to eventual loss of inhibitor while treating the fertilizer with the coating. For this reason, we used other methods to prove the sustained release effect of the physical film on N, NBPT, and DMPP. Firstly, we compared the SEM of CU3 and CSUs to clarify the effect of adding inhibitors on the envelope. Secondly, we compared the effects of CU3, SUs, and CSUs on soil N regulation ability, corn growth and yield to clarify the combined effect of coating, inhibitor, and coating + inhibitor. The SEM images of the surface and cross-section of CSU1 and CSU2 showed that spraying the inhibitor on the urea surface could improve the smoothness of the urea surface and make the film closely combined with urea (Figure 8D). Compared with CU3 (Figure 1D), the surface of the membrane shell of CSU2 was smooth, and the adhesion between the membrane shell and the urea surface was closer (Figures 8C,D). Combined with the CU3 nutrient release characteristic curve (Figure 2) and the scanning electron microscope test of CSUs (Figure 8), CSU1 and CSU2 could simultaneously realize the slow dissolution of N and inhibitors, and the controlled release performance of CSU2 was better than that of CSU1.

### Evaluation of the N Loss Potential and Soil Nitrogen Supply Capacity of the EENFs

Both NH_4_^+^-N and NO_3_^-^-N are the primary N forms absorbed by the plants (Zheng et al., 2017) and also the primary source of N loss (Xiao et al., 2019). After urea is applied to soil, it is rapidly hydrolyzed to (NH_4_)_2_CO_3_ under the soil urease, decomposition of the (NH_4_)_2_CO_3_ produces NH_4_^+^-N. The rapid hydrolysis of urea will cause a large amount of surplus NH_4_^+^-N, which is converted to NH_3_ (Adotey et al., 2017). However, this phenomenon provides sufficient substrate for nitrification so that soil nitrification is active (Xiao et al., 2019), resulting in the loss of NO_3_^-^-N, N_2_O, and N_2_. Urease and ammonia-oxidizing bacteria are the main factors of the soil N cycle. Inhibiting urease and ammonia-oxidizing bacteria can delay urea hydrolysis and transformation. Many studies have improved the duration of inhibitors by controlling the slow release of inhibitors. Li et al. (2020a) reported that the combined coating of HQ and urea could significantly reduce soil urease activity and prolong the action time of urease inhibitors during the growth period of wheat. Santos et al. (2020) found that the combined NBPT and biodegradable biofilm materials prepare controlled release urea, which could prolong the action time of NBPT and reduce the loss of N and NH_3_ volatilization. Zhang (2004) reported that the controlled dissolution of urea and DCD could significantly improve the nitrification inhibition effect. In this study, the results of the EENFs on NH_3_ volatilization loss and leaching loss were consistent with previous research conclusions. In addition, the leaching and NH_3_ volatilization tests were carried out without crop interference. The soil NH_3_ volatilization curves of SU1, SU2, CSU1, and CSU2 changed steadily throughout the culture stage (Figure 3). In the later stage of the leaching test, the leaching rate of NO_3_^-^-N of CSU1 and CSU2 was higher than that of SU1, SU2, and U (Figure 4). The changing trend of Figures 4E,F showed that the NBPT + DMPP could reduce N loss at the initial stage of fertilization, ensure sufficient N supply in the later stage of soil, which was also confirmed in the maize pot experiment (Figure 5). The results of the N loss potential clearly demonstrated that the urease and nitrification inhibitors effectively reduced N loss from urea, but their effectiveness weakened and N loss accelerated over time. Furthermore, compared with SU, CU3 was better in reducing N leaching loss, because the existence of the coating layer hinders the direct contact of nitrogen with the soil. When inhibitors treated urea were coated, both the fertilizer and the inhibitors were protected and the effective period of the inhibitors was prolonged. In addition, the inhibitors were more effective when sandwiched between the coating and the urea (CSU2) than when treated in the inner layer of the coating (CSU1).

Consistently, the higher availability of nutrients in the soil, the greater nutrients utilization by the crop plant (Alhaj Hamoud et al., 2019a). The change trend of inorganic N in maize pot experiment was found that different additional methods of NBPT + DMPP would affect soil N loss and soil N supply capacity. SU1 and SU2 reduced N leaching loss, while the accumulation of leaching loss increased rapidly in the later stage of culture as compared with the conventional urea. The leaching rate curves of CSU1 and CSU2 were stable during the culture period, these may be due to the physical film on the outer layer of the inhibitor, CSU1 and CSU2 could maintain the appropriate content of soil nutrients during the growth stage of maize (Figure 5), which might be due to CSU2 having two main regulatory effects on soil N transformation. Firstly, the physical film avoided the direct contact between urea and inhibitor and soil and controlled the slow release of urea and inhibitor. Secondly, the combination of NBPT and DMPP can, respectively, inhibit urease activity (Figure 6) and reduce the apparent nitrification rate of soil NH_4_^+^-N (Table 2), and regulate the transformation process of dissolving N. In addition, the results of the maize pot experiment showed that the NH_4_^+^-N in CSU2 soil reached the maximum at the heading stage of maize (Figure 5) to ensure sufficient N supply in the middle and late stage of maize growth.

### Effects of EENFs on Maize Yield and NUE

Crop yield and NUE are essential indicators for rational fertilization (Feng et al., 2020). The researchers (Yang et al., 2011; Geng et al., 2016; Zheng et al., 2016) reported that controlled-release urea met the long-term N demand of crops and improved crop yield and NUE. Li et al. (2020b) discussed that the combination of urea and HQ coated controlled-release urea could increase wheat yield by 56% compared with U. This experiment found that EENFs increased production significantly compared with conventional urea, consistent with the above report. Compared with U, CU3, SUs, and CSUs could promote the growth of maize and significantly increase maize yield and NUE 12–79% (Table 3) and 10–59% (Table 4), respectively. The growth stage of summer maize is from June to October each year, and the soil temperature and moisture are relatively high, which can accelerate the process of soil N conversion. Uncoated, stabilized EENFs (SUs) could regulate N transformation in the early stage of maize growth, however, the regulation ability of N delayed in the late stage of maize growth. In addition, CU3 could prevent the dissolution of N to a certain extent, but it was also greatly affected by the soil environment. The inhibitor and urea were co-coated (CSUs), and the hindering effect of the physical film layer could prolong the dissolution time of the inhibitor and N, and reduce the degradation and fixation of the inhibitor in the soil. The combined effect of coating and inhibitor strengthens the N supply capacity of the soil, guarantees effective nutrient supply in the middle and late stages of maize growth. Furthermore, the application of coated, stabilized EENFs in maize pot experiment was conducive to improve the matching degree between soil nutrient supply and maize nutrient demand, so that CSUs was better than SUs and CU3 in improving maize yield and NUE.

## Conclusion

Incorporation of NR in the ER coating reduces the usage of ER, whose accumulation in soil could be an environmental concern. When the ratio of ER to NR was 7:3, the NR-modified ER-coated urea presented good N release performance with a low first-day release and a long release period.

The incorporation method of urease and nitrification inhibitors significantly affected the N loss (NH_3_ volatilization, NH_4_^+^ and NO_3_^–^ leaching) and agronomic effectiveness of the CSUs. Compared with CSU1 where the inhibitors were homogeneously distributed in the innermost layer of the coating, less N was lost from CSU2 where the inhibitors were sandwiched between the fertilizer and the coating. In the treatment with CSU2 application of the pot experiment, soil NH_4_^+^ was low at the seedling and mature stages of maize but was high at the jointing, tasseling, and flowering stages, well matching the dynamic N demand of maize. Application of CSU2 significantly increased maize grain yield 27% and nitrogen use efficiency 9% as compared with CSU1 application. The results of this study provide a support for preparation of novel environmentally friendly coated, stabilized EENFs not only with low N loss and high NUE but also with less usage of ER.

## Data Availability Statement

The original contributions presented in the study are included in the article/supplementary material, further inquiries can be directed to the corresponding author/s.

## Author Contributions

YD: conceptualization, methodology, writing – reviewing and editing, and supervision. ZQ: data curation, writing – original draft preparation, software, and investigation. MW and YL: data curation, writing – original draft preparation, visualization, and investigation. MH: supervision. XD: software and validation. All authors contributed to the article and approved the submitted version.

## Conflict of Interest

The authors declare that the research was conducted in the absence of any commercial or financial relationships that could be construed as a potential conflict of interest.

## Publisher’s Note

All claims expressed in this article are solely those of the authors and do not necessarily represent those of their affiliated organizations, or those of the publisher, the editors and the reviewers. Any product that may be evaluated in this article, or claim that may be made by its manufacturer, is not guaranteed or endorsed by the publisher.
